# Supplementary material for: Structural insights into selective interaction between type IIa receptor protein tyrosine phosphatases and Liprin-α
Source: Nat Commun. 2020 Jan 31;11:649. doi: 10.1038/s41467-020-14516-5 (PMC6994669; doi:10.1038/s41467-020-14516-5)
Supplement: Supplementary file 1 — Supplementary Information [file 41467_2020_14516_MOESM1_ESM.pdf]

Supplementary Information

Structural insights into selective interaction between type IIa receptor protein tyrosine phosphatases and Liprin-α

Wakita et al.

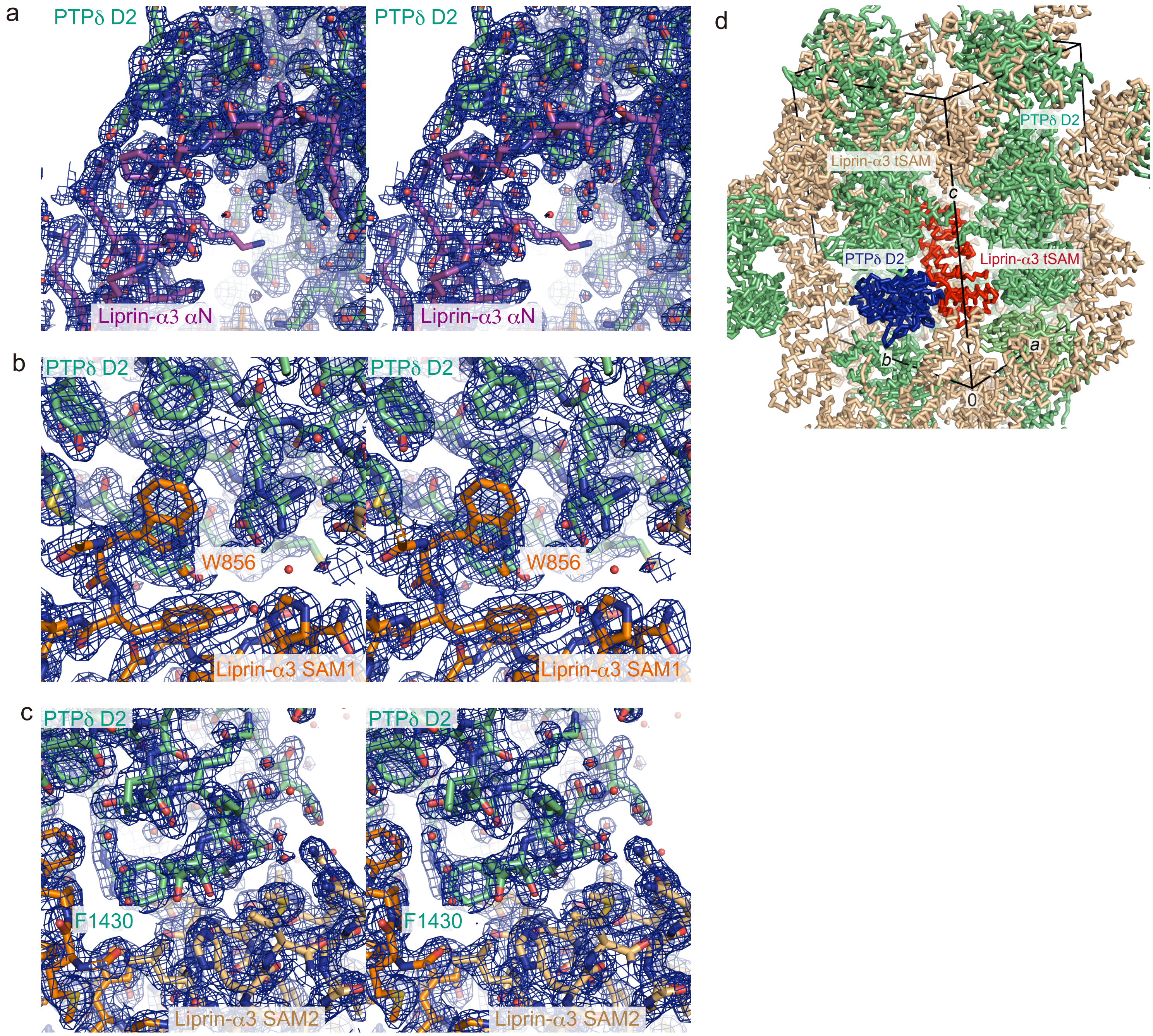

Supplementary Figure 1 Additional data of crystallography

(a–c) $2F_o$–F_c electron density maps of the interface between PTPδ D2 and Liprin-α3 tSAM coutoured at 1.0 σ (a) and 1.5 σ (b, c) level.

(d) Crystal packing of the complex of PTPδ D2 and Liprin-α3 tSAM. PTPδ D2 and Liprin-α3 tSAM in one complex is blue and red, respectively, and those in the other complexes (related by crystallographic symmetry) are green and light brown, respectively. The unit cell is drawn with black lines.

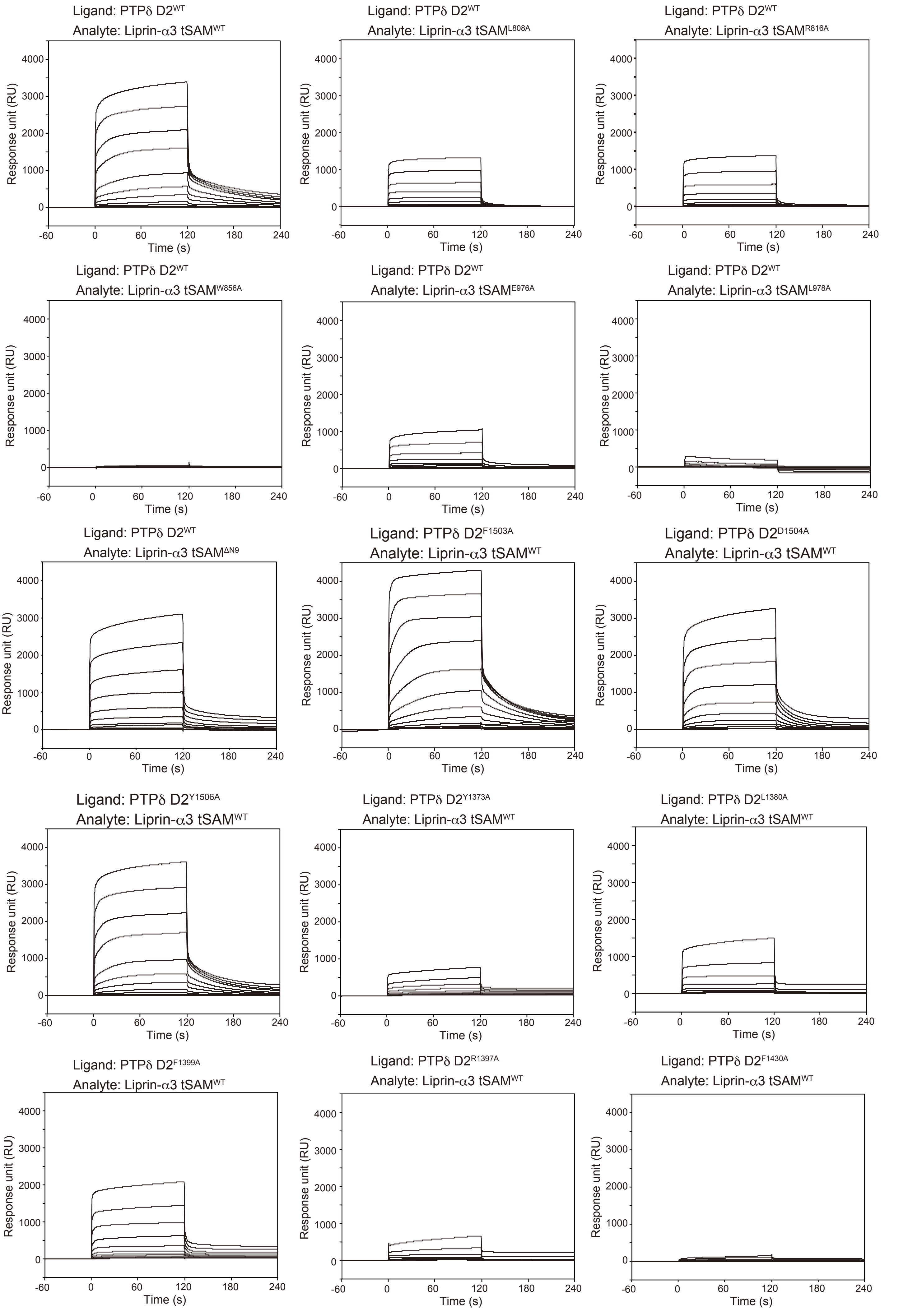

Supplementary Figure 2 SPR sensorgrams that were used to calculate the affinities shown in Table 2. One representative sensorgram from three independent measurements is shown for each ligand–analyte pair.

```
                                                                   Trp856
                              813                                               872
Mouse      Liprin-α3  DKDRRNKRKHELLEEACRQGLPFAAWDGPTVVSWLELWVGMPAWYVAACRANVKSGAIMA
                -α1  EKNRKLQKKHELLEEARRQGLPFAQWDGPTVVVWLELWVGMPAWYVAACRANVKSGAIMS
                -α2  EKDRRLKKKHELLEEARRKGLPFAQWDGPTVVAWLELWLGMPAWYVAACRANVKSGAIMS
                -α4  EKDRRLKKKHQLLEDARRKGMPFAQWDGPTVVSWLELWVGMPAWYVAACRANVKSGAIMS
Human      Liprin-α3  DKDRRNKRKHELLEEACRQGLPFAAWDGPTVVSWLELWVGMPAWYVAACRANVKSGAIMA
                -α1  EKNRKLQKKHELLEEARRQGLPFAQWDGPTVVVWLELWVGMPAWYVAACRANVKSGAIMS
                -α2  EKDRRLKKKHELLEEARRKGLPFAQWDGPTVVAWLELWLGMPAWYVAACRANVKSGAIMS
                -α4  EKDRRLKKKHQLLEDARRKGMPFAQWDGPTVVSWLELWVGMPAWYVAACRANVKSGAIMS
Rat        Liprin-α3  DKDRRNKRKHELLEEACRQGLPFAAWDGPTVVSWLELWVGMPAWYVAACRANVKSGAIMA
                -α1  EKNRKLQKKHELLEEARRQGLPFAQWDGPTVVVWLELWVGMPAWYVAACRANVKSGAIMS
                -α2  EKDRRLKKKHELLEEARRKGLPFAQWDGPTVVAWLELWLGMPAWYVAACRANVKSGAIMS
                -α4  EKDRRLKKKHQLLEDARRKGMPFAQWDGPTVVSWLELWVGMPAWYVAACRANVKSGAIMS
Xenopus    Liprin-α3  DKDRRSKKKHELLEEACRQGLPFASWDGPTVVSWLELWVGMPAWYVAACRANVKSGAIMA
                -α1  EKNRKLQKKHELLEEARRQGLPFAQWDGPTVVVWLELWVGMPAWYVAACRANVKSGAIMS
                -α2  EKDRRLKKKHELLEDARRKGLPFAQWDGPTVVAWLELWLGMPAWYVAACRANVKSGAIMS
Zebrafish  Liprin-α3  DKDRRSKKKHELLEEACRQGLPFASWDGPTVVSWLELWVGMPAWYVAACRANVKSGAIMA
                -α1  EKNRKLQKKHELLEEACRQGLPFAQWDGPTVVVWLELWVGMPAWYVAACRANVKSGAIMS
                -α2  EKDRRLKKKHELLEEARRKGLPFAQWDGPTVVAWLELWLGMPAWYVAACRANVKSGAIMS
                -α4  ERDRRMKKKHELLEDARRKGLPFAQWDGPTVVSWLELWVGMPAWYVAACRANVKSGAIMS
Drosophila Liprin-α   RQKKEHDYRNDLLGEAMKAGTPFALWNGPTIVAWLELWVGMPAWYVAACRANVKSGAIMS
Nematode   Liprin-α   DYDRRKKKKHELLEEAMKARTPFALWNGPTVVAWLELWVGMPAWYVAACRANVKSGAIMS

                      873                                                      932
Mouse      Liprin-α3  NLSDTEIQREIGISNPLHRLKLRLAIQEMVSLTSPSAPASSRTPTGNVWMTHEEMESLTA
                -α1  ALSDTEIQREIGISNPLHRLKLRLAIQEIMSLTSPSAPPTSRTTTGNVWLTHEEMETLTA
                -α2  ALSDTEIQREIGISNPLHRLKLRLAIQEMVSLTSPSAPPTSRTPSGNVWVTHEEMENLTA
                -α4  ALSDTEIQREIGISNALHRLKLRLAIQEMVSLTSPSAPPTSRTSSGNVWVTHEEMETLAT
Human      Liprin-α3  NLSDTEIQREIGISNPLHRLKLRLAIQEMVSLTSPSAPASSRTSTGNVWMTHEEMESLTA
                -α1  ALSDTEIQREIGISNPLHRLKLRLAIQEIMSLTSPSAPPTSRTT----------------
                -α2  ALSDTEIQREIGISNPLHRLKLRLAIQEMVSLTSPSAPPTSRTPSGNVWVTHEEMENLAA
                -α4  ALSDTEIQREIGISNALHRLKLRLAIQEMVSLTSPSAPPTSRTSSGNVWVTHEEMETLET
Rat        Liprin-α3  NLSDTEIQREIGISNPLHRLKLRLAIQEMVSLTSPSAPASSRTPTGNVWMTHEEMESLTA
                -α1  ALSDTEIQREIGISNPLHRLKLRLAIQEIMSLTSPSAPPTSRTT----------------
                -α2  ALSDTEIQREIGISNPLHRLKLRLAIQEMVSLTSPSAPPTSRTPSGNVWVTHEEMENLAA
                -α4  ALSDTEIQREIGISNALHRLKLRLAIQEMVSLTSPSAPPTSRTSSGNVWVTHEEMETLAT
Xenopus    Liprin-α3  NLSDTEIQREIGISNPLHRLKLRLAIQEMVSLTSPSAPASTRSSTSNVWMTHAEMESLTA
                -α1  ALSDTEIQREIGISNPLHRLKLRLAIQEIMSLTSPSAPATSRTT----------------
                -α2  ALSDTEIQREIGISNPLHRLKLRLAIQEMVSLTSPSAPPTSRTPSGNVWLTHEEMENLAA
Zebrafish  Liprin-α3  NLSDTEIQREIGISNPLHRLKLRLAIQEMVSLTSPSAPASTRSSTSNVWMTHAEMESLTA
                -α1  ALSDTEIQREIGISNPLHRLKLRLAIQEIMSLTSPSAPPTSRTT----------------
                -α2  ALSDTEIQREIGISNPLHRLKLRLAIQEMVSLTSPSAPPTSRTPSGNVWVTHEEMETMAA
                -α4  ALSDTEIQREIGISNPLHRLKLRLAIQEMVSLTSPSAPLTSRTSSGNVWVTHEEMENMAT
Drosophila Liprin-α   ALSDTEIQREIGISNPLHRLKLRLAIQEMVSLTSPSAPQTSRTT----------------
Nematode   Liprin-α   ALSDQEIQKEIGISNPLHRLKLRLAIQEMVSLTSPSAPRTARLT----------------

                                                                  Glu976
                      973                                  Arg971  Leu978      992
Mouse      Liprin-α3  ATKP-ETKEISWEQ------ILAYGDMNHEWVGNDWLPSLGLPQYRSYFMESLVDARMLD
                -α1  TPQTQEDEEGSWAQ------TLAYGDMNHEWIGNEWLPSLGLPQYRSYFMECLVDARMLD
                -α2  PAKTKESEEGSWAQCPVFLQTLAYGDMNHEWIGNEWLPSLGLPQYRSYFMECLVDARMLD
                -α4  STKT-DSEEGSWAQ------TLAYGDMNHEWIGNEWLPSLGLPQYRSYFMECLVDARMLD
Human      Liprin-α3  TTKP-ETKEISWEQ------ILAYGDMNHEWVGNDWLPSLGLPQYRSYFMESLVDARMLD
                -α1  ---------------------LAYGDMNHEWIGNEWLPSLGLPQYRSYFMECLVDARMLD
                -α2  PAKTKESEEGSWAQCPVFLQTLAYGDMNHEWIGNEWLPSLGLPQYRSYFMECLVDARMLD
                -α4  STKT-DSEEGSWAQ------TLAYGDMNHEWIGNEWLPSLGLPQYRSYFMECLVDARMLD
Rat        Liprin-α3  ATKP-ETKEISWEQ------ILAYGDMNHEWVGNDWLPSLGLPQYRSYFMESLVDARMLD
                -α1  ---------------------LAYGDMNHEWIGNEWLPSLGLPQYRSYFMECLVDARMLD
                -α2  PAKTKESEEGSWAQ------TLAYGDMNHEWIGNEWLPSLGLPQYRSYFMECLVDARMLD
                -α4  STKT-DSEEGSWAQ------TLAYGDMNHEWIGNEWLPSLGLPQYRSYFMECLVDARMLD
Xenopus    Liprin-α3  ATKP-ELKEISWDQ------ILAYGDMNHEWVGNDWLPSLGLPQYRSYFMESLVDARMLD
                -α1  ---------------------LAYGDMNHEWIGNEWLPSLGLPQYRSYFMECLVDARMLD
                -α2  PSKTKESEEGSWAQ------TLAYGDMNHEWIGNEWLPSLGLPQYRSYFMECLVDARMLD
Zebrafish  Liprin-α3  ATKP-ELKEISWDQ------ILAYGDMNHEWVGNDWLPSLGLPQYRSYFMESLVDARMLD
                -α1  ---------------------LAYGDMNHEWIGNDWLPSLGLPQYRSYFMESLVDARMLD
                -α2  PSKTSDSEEGSWSQ------TLAYGDMNHEWIGNEWLPSLGLPQYRSYFMECLVDARMLD
                -α4  STKM-DNEEGSWAQ------TLAYGDMNHEWIGNEWLPSLGLPQYRSYFMECLVDARMLD
Drosophila Liprin-α   ---------------------LAFGDMNHEWIGNYWLPGLGLPQYRTTFMECLVDARMLD
Nematode   Liprin-α   ---------------------LAFGDMNHEYIGNDWLPCLGLAQYRSAFMECLLDARMLE
```

αN SAM1 SAM2

Supplementary Figure 3 Sequence alignment of the IIa RPTP-binding regions of Liprin-α family proteins.

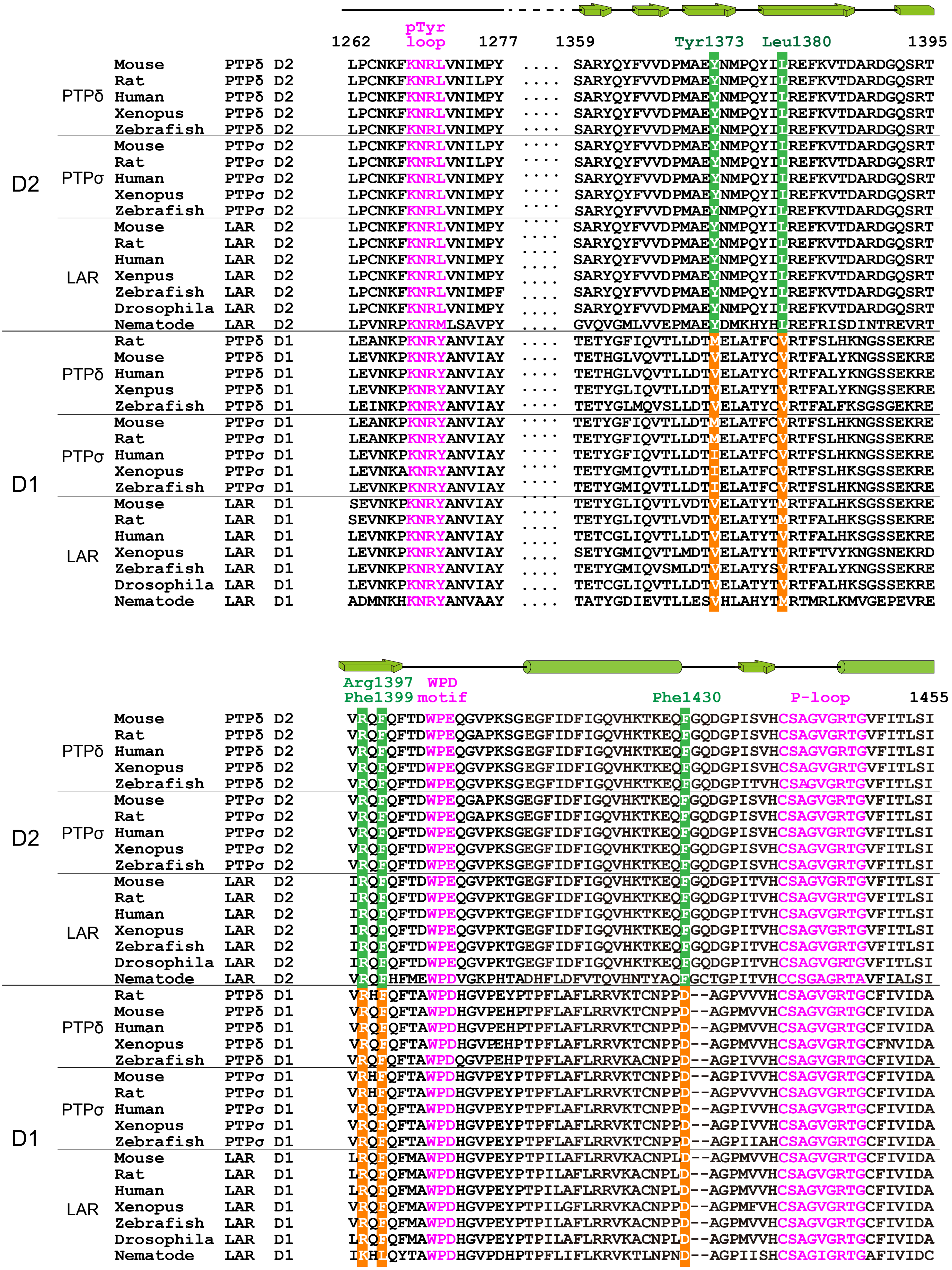

Supplementary Figure 4 Sequence alignment of the (pseudo-)catalytic sites and Liprin-α-binding regions of IIa RPTPs.

```
                                 835                        Trp856                                892
Mouse       Liprin-α3 FAAWDGPTVVSWLELWVGMPAWYVAACRANVKSGA--IMANLSDTEIQREIGISNPLHRL
                  -α1 FAQWDGPTVVVWLELWVGMPAWYVAACRANVKSGA--IMSALSDTEIQREIGISNPLHRL
                  -α2 FAQWDGPTVVAWLELWLGMPAWYVAACRANVKSGA--IMSALSDTEIQREIGISNPLHRL
                  -α4 FAQWDGPTVVSWLELWVGMPAWYVAACRANVKSGA--IMSALSDTEIQREIGISNALHRL
Mouse       Liprin-β1 FAKWTKEQVCSWLAEQG--LGSYLSSGKHWIISGQ--TLLQASQQDLEKELGIKHSLHRK
                  -β2 FAQWSTERVCTWMEDFG--LGQYVIFARQWVTSGH--TLLTATPQDMEKELGIKHPLHRK
Human       Liprin-β1 FAKWTKEQVCNWLMEQG--LGSYLNSGKHWIASGQ--TLLQASQQDLEKELGIKHSLHRK
                  -β2 FAQWSTERVCAWLEDFG--LAQYVIFARQWVSSGH--TLLTATPQDMEKELGIKHPLHRK
Rat         Liprin-β1 FAKWTKEQVCSWLAEQG--LGSYLSSGKHWIMSGQ--TLLQASQQDLEKELGIKHSLHRK
                  -β2 FAQWSTERVCTWLQDFG--LAQYVIFARQWVASGH--TLLTATPQDMEKELGIKHPLHRK
Xenopus     Liprin-β1 FAKWTKDQVCSWLRDQG--LGGYVSSCKQWIVSGQ--TLLHASQHDLEKELGIKQPLHRK
                  -β2 FAKWTKDQVCSWLRDQG--LGGYVNSCKQWIVSGQ--TLLHASQQDLEKELGIKQPLHRK
Zebrafish   Liprin-β1 FARWSREQVCDWMQEQG--LGLYVTLARQWVSSGQ--TLLQASQHDLERELGIKHPLHRK
                  -β2 FSKWSCDQVCTWMEDFG--LGQYVNMARQWVTSGQ--SLLSASSHEIEKELGIKHPLHRK
Drosophila  Liprin-β  WTTWGAQEVCNWLAYMG--LGCYEDNCRKWLNANPSVSFFTASPVDIERELNLKMPLHRK
Nematode    Liprin-β  FVDWRSEQLADWIAEIG--YPQYMNEVSRHVRSGR--HFLNMSMNEYEGVLNIKNPVHRK

                      893                                                   945
Mouse       Liprin-α3 KLRLAIQEMVSLTSPSAPASSRTPTGNVWMTHEEMESLTAATKP-ETKEISWEQ------
                  -α1 KLRLAIQEIMSLTSPSAPPTSRTTTGNVWLTHEEMETLTATPQTQEDEEGSWAQ------
                  -α2 KLRLAIQEMVSLTSPSAPPTSRTPSGNVWVTHEEMENLTAPAKTKESEEGSWAQCPVFLQ
                  -α4 KLRLAIQEMVSLTSPSAPPTSRTSSGNVWVTHEEMETLATSTKT-DSEEGSWAQ------
Mouse       Liprin-β1 KLQLALQALGSEEET---------------------------------------------
                  -β2 KLVLAVKAINAKQEE---------------------------------------------
Human       Liprin-β1 KLQLALQALGSEEET---------------------------------------------
                  -β2 KLVLAVKAINTKQEE---------------------------------------------
Rat         Liprin-β1 KLQLALQALGSEEET---------------------------------------------
                  -β2 KLVLAVKAINTKQEE---------------------------------------------
Xenopus     Liprin-β1 KLQLALQSLGSEDES---------------------------------------------
                  -β2 KLQLALQSLGSEDES---------------------------------------------
Zebrafish   Liprin-β1 KLQLALQALGSEEDD---------------------------------------------
                  -β2 KLQLALHSFSSKITE---------------------------------------------
Drosophila  Liprin-β  KILLAIDDLTGKEFDDL-------------------------------------------
Nematode    Liprin-β  RVAILLRRIEEDIMEP--------------------------------------------

                                                        Glu976
                      946                        Arg971 Leu978                    1005
Mouse       Liprin-α3 ILAYGDMNHEWVGNDWLPSLGLPQYRSYFMESLVDARMLDHLNKKELRGQLKMVDSFHRV
                  -α1 TLAYGDMNHEWIGNEWLPSLGLPQYRSYFMECLVDARMLDHLTKKDLRGQLKMVDSFHRN
                  -α2 TLAYGDMNHEWIGNEWLPSLGLPQYRSYFMECLVDARMLDHLTKKDLRVHLKMVDSFHRT
                  -α4 TLAYGDMNHEWIGNEWLPSLGLPQYRSYFME LVDARMLDHLTKKDLRVHLKMVDSFHRT
Mouse       Liprin-β1 --NYGKLDFNWVT-RWLDDIGLPQYKTQFDEGRVDGRMLHYMTVDDL-LSLKVVSVLHHL
                  -β2 --TSALLDHIWVT-RWLDDIGLPQYKDQFHESRVDGRMLQYLTVNDL-LFLKVTSQLHHL
Human       Liprin-β1 --NHGKLDFNWVT-RWLDDIGLPQYKTQFDEGRVDGRMLHYMTVDDL-LSLKVVSVLHHL
                  -β2 --KSALLDHIWVT-RWLDDIGLPQYKDQFHESRVDGRMLQYLTVNDL-LFLKVTSQLHHL
Rat         Liprin-β1 --NYGKLDFNWVT-RWLDDIGLPQYKTQFDEGRVDGRMLHYMTVDDL-LSLKVVSVLHHL
                  -β2 --KSALLDHVWVT-RWLDDIGLPQYKDQFYESRVDGRMLQYLTVNDL-LFLKVTSQLHHL
Xenopus     Liprin-β1 --NYGRLDYRWVT-RWLDDIGLPQYKTQFDDSKIDGRMLHYLAVEDL-LSLKVVSVLHHL
                  -β2 --NYGRLDYRWVT-RWLDDIGLPQYKTQFDDAKLDGRMLHYLAVEDL-LSLKVVSVLHHL
Zebrafish   Liprin-β1 --NKGKLDYHWVT-RWLDDIGLPQYKTQFDEGRVDGRMLHYMTVDDL-LSLKVGSVLHHL
                  -β2 --KSSELDYIWVT-RWLDDIGLPQYKDQFNEGRVDGRMLQYLTVNDL-LFLKVTSQLHHL
Drosophila  Liprin-β  TLKASSLDVTWVL-RWLDDIGLPQYKDYFMQAKIDGRMLHRLTLEDL-SQLHVSSCLHIA
Nematode    Liprin-β  ---ANKWDVHQTL-RWLDDIGLPQYKDVFAENVVDGPLLLSLTANDA-VEMKVVNAHHYA
```

Supplementary Figure 5 Sequence alignment of the IIa RPTP-binding region of Liprin-α family proteins and the corresponding region of and Liprin-β family proteins.

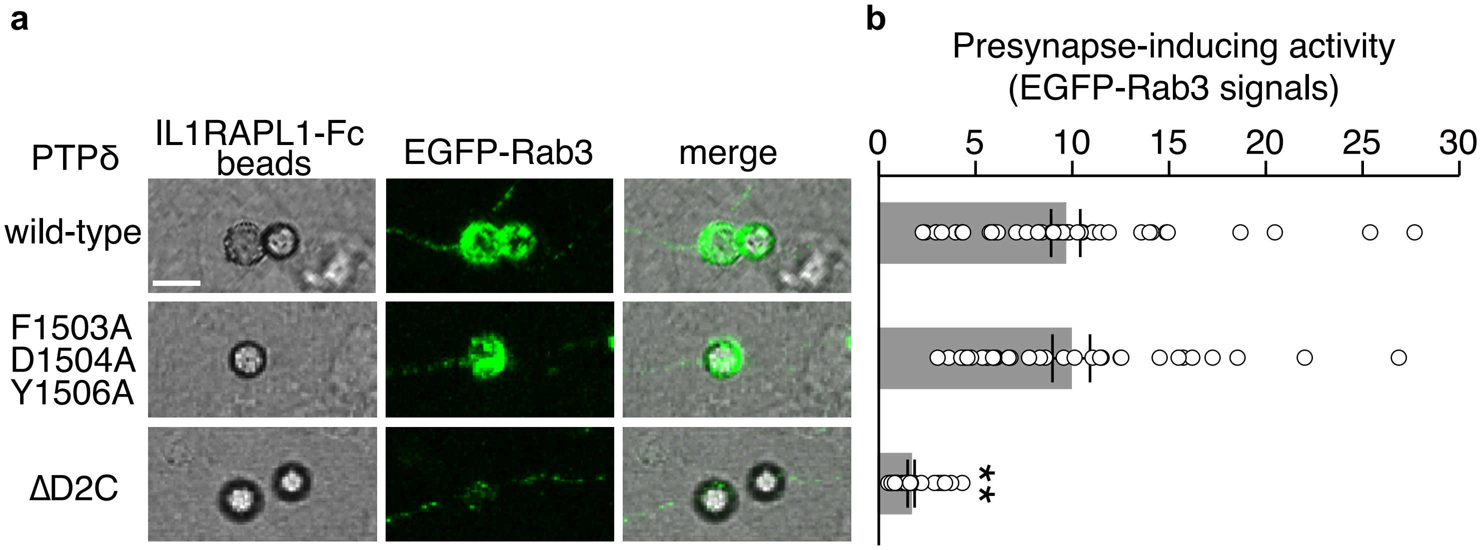

Supplementary Figure 6 Synaptogenic activitiy of a PTPδ mutant for the interface with αN of Liprin-α3 tSAM.

(a) Rescue of IL1RAPL1-induced EGFP-Rab3 accumulation in *Ptprd*$^{meB*/meB*}$ cortical neurons by transient expression of wild-type and mutated forms of PTPδ.

(b) Relative intensity of EGFP-Rab3 signals on the surface of beads in (a) (n = 49, 34, and 33 beads for wild-type, F1503A/D1504A/Y1506A, and ΔD2C, respectively). All values represent mean ± SEM. **, P < 0.01 compared wild-type PTPδ; Tukey' s test. Scale bar, 5 μm.

Source data are provided as a Source Data file.

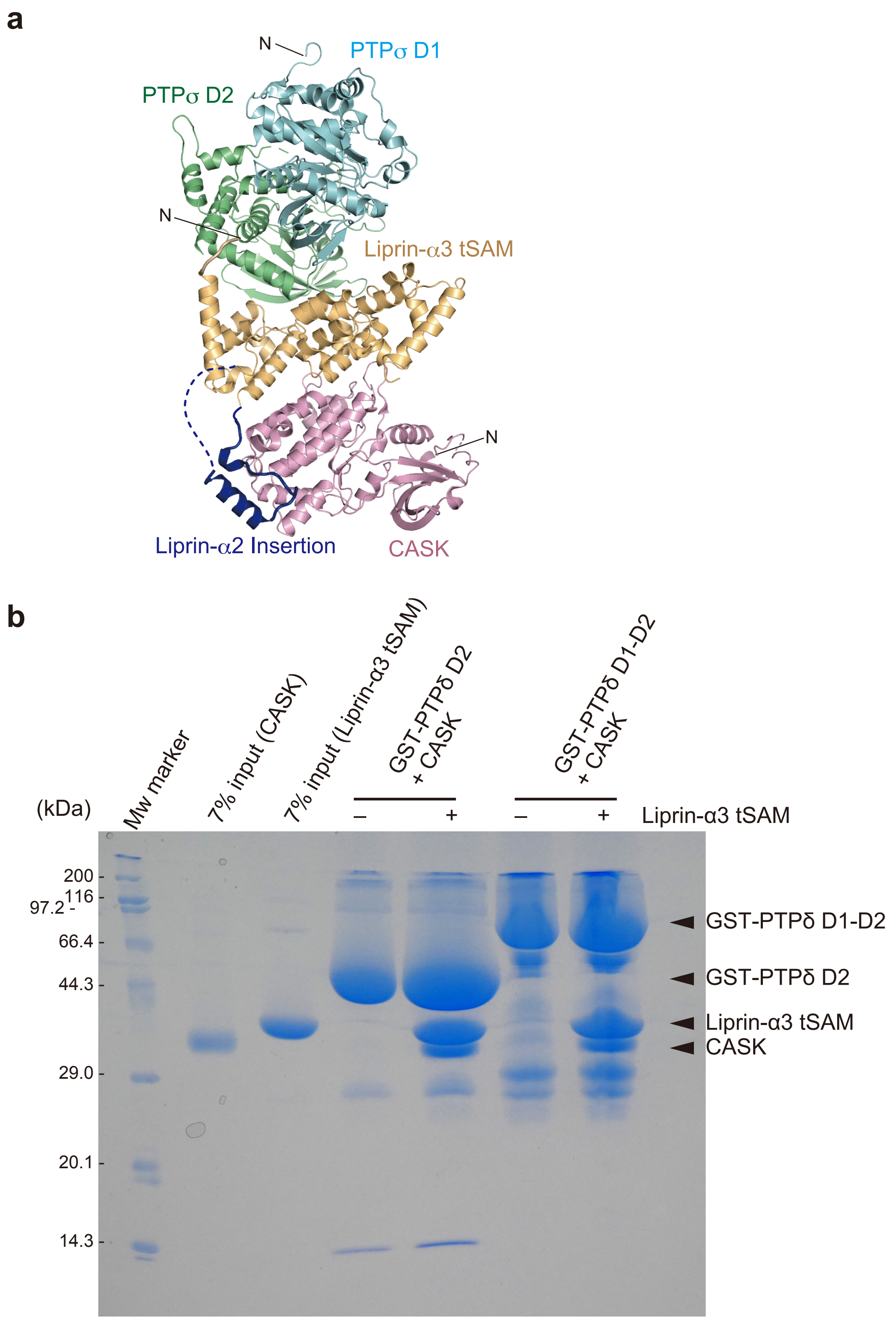

Supplementary Figure 7 Tripartite assembly of IIa RPTP D1-D2, Liprin-α, and CASK

(a) A model of the tripartite complex composed of IIa RPTP D1-D2, Liprin-α tSAM and CASK, based on the superposition of PTPσ D1-D2 (D1, cyan; D2, green) onto the PTPδ D2–Liprin-α–CASK complex shown in Fig. 5b. Coloring scheme of Liprin-α and CASK is the same as that in Fig. 5b.

(b) GST-pulldown assay for analyzing the formation of the tripartite complex suggested in (a). Proteins bound to GST-PTPδ D2 and D1-D2 were subjected to 14% SDS-PAGE and stained with Coomassie Brilliant Blue. Source data are provided as a Source Data file.

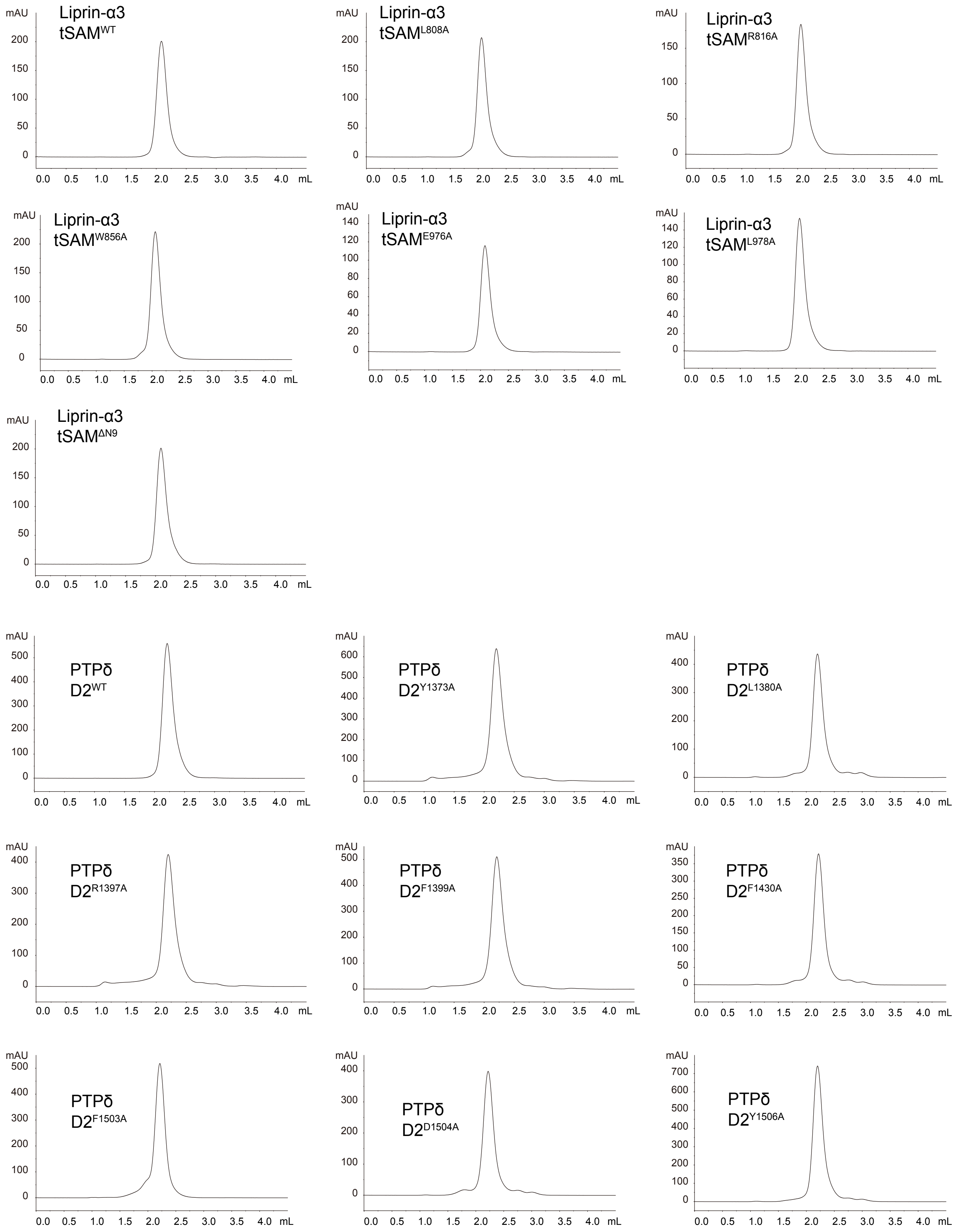

Suppelentary Figure 8 Size-exclusion chromatography of wild-type and mutant Liprin-α3 tSAM and PTPδ D2 proteins.

Samples used for SPR experiments were analyzed by a Superdex 200 5/150 column with 20 mM Tris-Cl (pH 7.5) containing 150 mM NaCl.

Supplementary Table 1 Additional information for rmsd calculations

Reference (residue range)	Target	PDB ID	Domain (residue range)	Chain ID	Cα rmsd (Å)	Residues used for calculation
PTPδ D2 (1213–1507)	PTPδ	2NV5	D1 (1204–1486)	A	1.10	272
				B	1.10	278
				C	1.10	271
	PTPσ	2FH7	D1 (1367–1649)	A	1.23	270
			D2 (1650–1942)	A	0.59	283
	LAR	1LAR	D1 (1307–1584)	A	1.22	265
			D1 (1340–1584)	B	1.11	244
			D2 (1585–1876)	A	0.83	287
				B	0.95	287
Liprin-α3 tSAM (807–1114)	Liprin-α2	3TAC	tSAM (871–1182)	B	1.39	258

Supplementary Table 2 Summary of the analysis of the interface between Liprin-α3 tSAM and PTPδ D2.

Interface	Buried surface area (Å^2)	Interaction	Liprin-α3 tSAM	PTPδ D2
αN	443	vdw	LYS 807	TYR1462
		Hydrophobic	**LEU 808**	**PHE1503**
		Hydrophobic	**LEU 808**	TYR1506
		Hydrophobic	**LEU 808**	LEU1500
		Hydrogen bond	**LEU 808** [N]	ARG1461 [O]
		Hydrogen bond	THR 809 [N]	**TYR1506 [OH]**
		Hydrogen bond	THR 809 [OG1]	**TYR1506 [OH]**
		Hydrogen bond	THR 809 [O]	HIS1505 [ND1]
		Hydrogen bond	THR 809 [OG1]	HIS1505 [ND1]
		vdw	GLY 810	HIS1505
		Hydrogen bond	PRO 811 [O]	**ASP1504 [N]**
		vdw	GLY 812	PHE1503
		Hydrogen bond	ASP 813 [N]	SER1502 [O]
		Hydrogen bond	**ARG 816 [NH1]**	**ASP1504 [OD1]**
		Hydrogen bond	**ARG 816 [NH2]**	**ASP1504 [OD2]**
SAM1	369	Hydrophobic	PRO 854	**PHE1430**
		vdw	ALA 855	GLN1422
		Hydrophobic	**TRP 856**	**PHE1399**
		Hydrophobic	**TRP 856**	**LEU1380**
		Hydrophobic	**TRP 856**	**TYR1373**
		Hydrophobic	**TRP 856**	TYR1378
		Hydrophobic	**TRP 856**	GLU1382
		vdw	ALA 859	MET1375
		Hydrogen bond	GLY 884 [O]	**TYR1373 [OH]**
		Hydrophobic	HIS 890	**PHE1430**
SAM2	349	Hydrogen bond	HIS 954 [NE2]	GLN1429 [O]
		Hydrogen bond	GLU 955 [OE1]	GLN1429 [NE2]
		Hydrogen bond	ARG 971 [NH1]	ASP1433 [OD1]
		vdw	SER 972	GLY1431
		vdw	SER 972	GLN1432
		vdw	MET 975	GLY1431
		Hydrogen bond	**GLU 976 [N]**	**ARG1397 [NH1]**
		vdw	**GLU 976**	THR1395
		vdw	**GLU 976**	**PHE1430**
		Hydrophobic	**LEU 978**	**PHE1430**
		Hydrogen bond	LYS 997 [NZ]	SER1393 [O]

vdw, van der Waals interaction; mutated residues in this study are indicated by bold fonts.

The interface was first analyzed by the program PISA (http://www.ebi.ac.uk/pdbe/prot_int/pistart.html), and the result was further checked by manual inspection of the atomic model and electron density.
