# Supplementary material for: Structural insights into selective interaction between type IIa receptor protein tyrosine phosphatases and Liprin-α
Source: Nat Commun. 2020 Jan 31;11:649. doi: 10.1038/s41467-020-14516-5 (PMC6994669; doi:10.1038/s41467-020-14516-5)
Supplement: Supplementary file 3 — Reporting Summary [file 41467_2020_14516_MOESM3_ESM.pdf]

nature research

Corresponding author(s): Shuya Fukai, Tomoyuki Yoshida

Last updated by author(s): Jan 6, 2020

Reporting Summary

Nature Research wishes to improve the reproducibility of the work that we publish. This form provides structure for consistency and transparency in reporting. For further information on Nature Research policies, see Authors & Referees and the Editorial Policy Checklist .

Statistics

For all statistical analyses, confirm that the following items are present in the figure legend, table legend, main text, or Methods section.

n/a	Confirmed	
☐	☒	The exact sample size (*n*) for each experimental group/condition, given as a discrete number and unit of measurement
☐	☒	A statement on whether measurements were taken from distinct samples or whether the same sample was measured repeatedly
☐	☒	The statistical test(s) used AND whether they are one- or two-sided<br>*Only common tests should be described solely by name; describe more complex techniques in the Methods section.*
☐	☒	A description of all covariates tested
☒	☐	A description of any assumptions or corrections, such as tests of normality and adjustment for multiple comparisons
☐	☒	A full description of the statistical parameters including central tendency (e.g. means) or other basic estimates (e.g. regression coefficient) AND variation (e.g. standard deviation) or associated estimates of uncertainty (e.g. confidence intervals)
☐	☒	For null hypothesis testing, the test statistic (e.g. *F*, *t*, *r*) with confidence intervals, effect sizes, degrees of freedom and *P* value noted<br>*Give P values as exact values whenever suitable.*
☒	☐	For Bayesian analysis, information on the choice of priors and Markov chain Monte Carlo settings
☒	☐	For hierarchical and complex designs, identification of the appropriate level for tests and full reporting of outcomes
☒	☐	Estimates of effect sizes (e.g. Cohen's *d*, Pearson's *r*), indicating how they were calculated

Our web collection on statistics for biologists contains articles on many of the points above.

Software and code

Policy information about availability of computer code

Data collection	Crystallographic data collection: BSS (SPring-8 BL41XU)<br>SPR analysis: Biacore T200 software
Data analysis	Crystallographic data processing: HKL2000, CCP4, Coot, Phenix<br>SPR analysis: Biacore T200 software<br>Statistical analysis: Statcel2

For manuscripts utilizing custom algorithms or software that are central to the research but not yet described in published literature, software must be made available to editors/reviewers. We strongly encourage code deposition in a community repository (e.g. GitHub). See the Nature Research guidelines for submitting code & software for further information.

Data

Policy information about availability of data

All manuscripts must include a data availability statement. This statement should provide the following information, where applicable:

- Accession codes, unique identifiers, or web links for publicly available datasets
- A list of figures that have associated raw data
- A description of any restrictions on data availability

Data supporting the findings of this manuscript are available from the corresponding authors upon reasonable request. The coordinates and structure factors of the PTPδ D2–Liprin-α3 tSAM complex have been deposited in the Protein Data Bank with the accession code 6KIP. The uncropped gel images for Fig. 5c and Supplementary Fig. 7c are provided as a Source Data file. Other data are available from the corresponding authors upon reasonable request

Field-specific reporting

Please select the one below that is the best fit for your research. If you are not sure, read the appropriate sections before making your selection.

- [x] Life sciences
- [ ] Behavioural & social sciences
- [ ] Ecological, evolutionary & environmental sciences

For a reference copy of the document with all sections, see nature.com/documents/nr-reporting-summary-flat.pdf

Life sciences study design

All studies must disclose on these points even when the disclosure is negative.

Sample size
No sample-size calculations were performed. However, our sample sizes are comparable with or larger than those previously reported studies with similar experimental designs.

Data exclusions
No data excluded.

Replication
Data were collected from at least two separate experiments.

Randomization
No randomization.

Blinding
For synaptogenic assays, bead-neuron cocultures were randomly imaged in a blinded manner with regard to the expression vectors transfected and genotype of neurons.

Reporting for specific materials, systems and methods

We require information from authors about some types of materials, experimental systems and methods used in many studies. Here, indicate whether each material, system or method listed is relevant to your study. If you are not sure if a list item applies to your research, read the appropriate section before selecting a response.

Materials & experimental systems

n/a	Involved in the study	
x		Antibodies
x		Eukaryotic cell lines
x		Palaeontology
	x	Animals and other organisms
x		Human research participants
x		Clinical data

Methods

n/a	Involved in the study	
x		ChIP-seq
x		Flow cytometry
x		MRI-based neuroimaging

Animals and other organisms

Policy information about studies involving animals; ARRIVE guidelines recommended for reporting animal research

Laboratory animals
Mice on a C57BL/6N background were used. All the animal experiments were approved by the Animal Experiment Committee of the University of Toyama and conducted in accordance with the Guidelines for the Care and Use of Laboratory Animals of the University of Toyama.

Wild animals
n/a

Field-collected samples
n/a

Ethics oversight
n/a

Note that full information on the approval of the study protocol must also be provided in the manuscript.
